# Quantitative Limits on Small Molecule Transport via the Electropermeome — Measuring and Modeling Single Nanosecond Perturbations

**DOI:** 10.1038/s41598-017-00092-0

**Published:** 2017-03-03

**Authors:** Esin B. Sözer, Zachary A. Levine, P. Thomas Vernier

**Affiliations:** 1 Frank Reidy Research Center for Bioelectrics, Old Dominion University, Norfolk, VA 23508 USA; 2 Department of Physics, Department of Chemistry and Biochemistry, University of California Santa Barbara, Santa Barbara, CA 93106 USA

## Abstract

The detailed molecular mechanisms underlying the permeabilization of cell membranes by pulsed electric fields (electroporation) remain obscure despite decades of investigative effort. To advance beyond descriptive schematics to the development of robust, predictive models, empirical parameters in existing models must be replaced with physics- and biology-based terms anchored in experimental observations. We report here absolute values for the uptake of YO-PRO-1, a small-molecule fluorescent indicator of membrane integrity, into cells after a single electric pulse lasting only 6 ns. We correlate these measured values, based on fluorescence microphotometry of hundreds of individual cells, with a diffusion-based geometric analysis of pore-mediated transport and with molecular simulations of transport across electropores in a phospholipid bilayer. The results challenge the “drift and diffusion through a pore” model that dominates conventional explanatory schemes for the electroporative transfer of small molecules into cells and point to the necessity for a more complex model.

## Introduction

Electropulsation (electroporation, electropermeabilization) technology is widely used to facilitate transport of normally impermeant molecules into cells. Applications include electrochemotherapy^1^, gene electrotransfer therapy^2^, calcium electroporation^3^, electroablation^4^, food processing^5^, and waste-water treatment^6^. Even after 50 years of study, however, protocols for these applications depend to a large extent on empirical, operationally determined parameters. To optimize existing procedures and develop new ones, to provide practitioners with methods and dose-response relationships specific for each application, a predictive, biophysics-based model of electropermeabilization is needed. By definition, such a model must represent accurately the movement of material across the cell membrane. Validation of this key feature requires quantitative measurements of electroporative transport.

Electrophysical models^7, 8^ have guided electropulsation studies from the beginning. More recently, molecular dynamics (MD) simulations^9–12^ have helped to clarify the physical basis for the electroporation of lipid bilayers. Continuum models contain many empirical “fitting” parameters^13, 14^ and therefore are not accurately predictive for arbitrary systems. MD simulations provide a physics-based view of the biomolecular structures associated with electropermeabilization but are presently restricted for practical reasons to very short time (<1 ms) and distance (<1 µm) scales. Ongoing technological advances will overcome the computational resource barriers, enabling a synthesis of continuum and molecular models that will provide a solid foundation for a predictive, multi-scale model, but only if the assumptions and approximations associated with these models can be verified by comparison with relevant experimental data.

Most published observations of small molecule transport across membranes are either qualitative descriptions of the time course of the uptake of fluorescent dyes extracted from images of individual cells or more or less quantitative estimates or measurements of uptake into cell populations based on flow cytometry, fluorescence photomicrography, analytical chemistry, or cell viability. In two of these studies quantitative transport data were extracted from images of individual cells captured over time, providing information about the rate of uptake, the spatial distribution of the transport, and the variation among cells in a population^15, 16^. One of these reports^15^, however, describes transport after exposures to long (40 µs) pulses, which complicates the interpretation of the results, since the cellular response to electropulsation begins on a much shorter time scale.

After the development of a porating transmembrane potential^17^, some or all of the following may occur: normally impermeant material begins to cross the membrane^18, 19^, membrane conductivity greatly increases^20^, the resting transmembrane potential decreases^21^, phosphatidylserine is externalized^22^, osmotic balance is disrupted^21, 23^, lipids are peroxidized^24, 25^, ATP and K^+^ leak into the extracellular medium^26–28^ Ca^2+^ enters the cell^29, 30^, and membrane proteins may be electroconformationally altered^31^. Each of these events alone represents a significant physiological perturbation. Taken together they present a serious assault on the physical and biochemical integrity of the cell, which responds immediately by initiating membrane repair^32^ and the restoration of ion gradients and osmotic balance^33^—highly energy-intensive processes. Longer pulses and multiple pulses act on a transformed target, no longer an intact cell with normal physiology but a perturbed cell with draining resources attempting to repair damage and re-establish homeostatic equilibrium.

The stochastic pore model^7, 8^ dominates commonly accepted mechanistic schemes for electroporative transport of ions and small molecules and is consistent at least in broad outline with MD representations of lipid pores. Although it has been established that pulsed electric-field-driven uptake of plasmid DNA is a multi-step process that involves membrane restructuring beyond the formation of simple electropores^34^, it is generally assumed that the small fluorescent dye molecules commonly used as indicators of membrane permeabilization enter cells through lipid electropores^16, 35^ like those in the models^36, 37^. Because electroporated cell membranes remain permeable for many seconds and even minutes after pulse delivery^26, 38^, electrophoresis of charged species through electropores during pulse application (fractions of a second) can be only a small fraction of the net uptake. Post-pulse diffusion through long-lived pores must dominate transport in these models. Our results challenge this conventional picture of electroporative transport of small molecules into cells.

In the work reported here, we use single, very short pulses that last roughly the amount of time it takes to form a lipid electropore^9, 11, 12^. By minimizing the permeabilizing electric field exposure and thereby limiting the cascade of secondary consequences, we narrow our focus to effects resulting from the immediate interactions of the electric field with the cell. Single-short-pulse permeabilization reduces the confounding factors arising from longer pulses, where the field continues to be applied after the membrane is already permeabilized, and from multiple pulses, where the field is applied to cells that are already responding to the disruptions to homeostasis resulting from permeabilization by the initial pulse.

Specifically, we provide a quantitative, single-cell-based description of the time course of uptake of the fluorescent dye YO-PRO-1 (YP1)^18^ into human lymphoid cells (U-937) permeabilized by a single 6 ns, 20 MV/m electric pulse. We determine not only the molecular rate of entry of YP1 but also the extent of uptake for each cell and the cell-to-cell variation. We compare these measurements with molecular dynamics (MD) simulations of YP1 interactions with a phospholipid bilayer in a permeabilizing electric field.

The results show that even after this single brief pulse the uptake continues at least for ten minutes. Moreover, there is a great heterogeneity in response among the cell population (n = 157) that has no correlation with the cell size. The investigations with molecular dynamics simulations point to binding of the dye significantly with the lipid bilayer, and that transport of the molecule occurs not in a free-flowing manner through the electropore but while dye stays closely associated with the pore walls. Related experimental data also indicate this dye is adsorbed significantly by the membrane. The diffusion-based calculations of the transport give results with large uncertainties, which can accommodate some of the end results but cannot model complex interactions of the transport molecule with the membrane and subsequent long-lasting biological responses that are triggered by electropulsation. We suggest improving current electroporation models by including structures and processes that contribute to electropermeabilization, which, as a whole, we call the *electropermeome*.

## Results

### Single, 6 ns pulse-induced uptake of YO-PRO-1 by U-937 human histiocytic lymphoma cells

**Figure 1:**
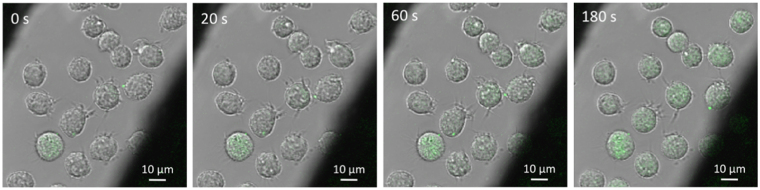
YO-PRO-1 uptake by U-937 cells at 0 s, 20 s, 60 s, and 180 s after delivery of a single, 6 ns, 20 MV/m pulse. Overlay of representative transmitted and fluorescence confocal images. The dark areas at upper left and lower right are the pulse generator electrodes.

**Figure 2:**
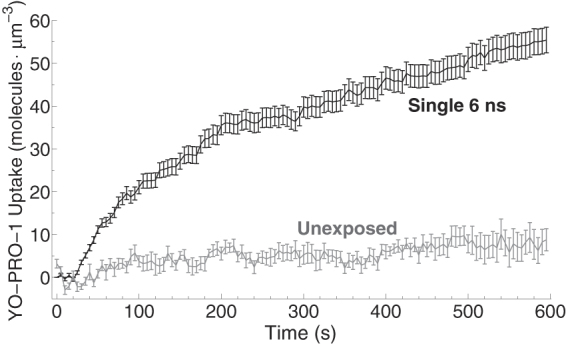
Uptake of YP1 after application of a single, 6 ns, 20 MV/m electric pulse delivered at t = 22 s. Control (unexposed) cells from the same experiments (n = 10) exhibit very much slower uptake. After an initial rate of about 180 YP1 · cell^−1^ · s^−1^, net uptake slows to nearly zero at about 200 s, then resumes at a slower rate, about 25 YP1 · cell^−1^ · s^−1^ (n = 157).

Quantitative fluorescence microphotometric analysis^16^ of observations of single U-937 cells (Fig. 1) reveals the kinetics of YO-PRO-1 (YP1) uptake after a single 6 ns permeabilizing electric pulse (Fig. 2). This minimal exposure reduces the confounding factors arising from longer pulses, where the field continues to be applied after the membrane is already permeabilized, and from multiple pulses, where the field is applied to cells that are already responding to the disruptions to homeostasis resulting from permeabilization by the initial pulse. A 6 ns, 20 MV/m permeabilizing event induces YP1 uptake for at least 10 minutes with cells in growth medium (RPMI-1640) containing 2 µM YP1 (Fig. 2).

Over the first 20 seconds after pulse delivery intracellular YP1 concentration increases by about 7 YP1 · µm^−3^ (12 nM)—about 180 molecules per cell per second. Given that U-937 cells are roughly spherical, with an average radius of 5 µm (*A*_*cell*_ ≈ 310 µm^2^), and that YP1 entry under these conditions is distributed uniformly around the cell perimeter (data not shown), the uptake per unit area is 0.6 YP1 · s^−1^ · µm^2^. Transport slows to 25 YP1 s^−1^ · cell^−1^ after three minutes and continues at that lower rate, resulting in a total of about 40,000 YP1 transported, on average, into each cell in 10 minutes. These numbers are roughly comparable to the 5 YP1 · µm^−3^ per pulse reported for YP1 uptake in a different cell type (CHO) after a different pulse exposure—longer duration but lower electric field (60 ns, 1.3 MV/m)^16^.

**Figure 3:**
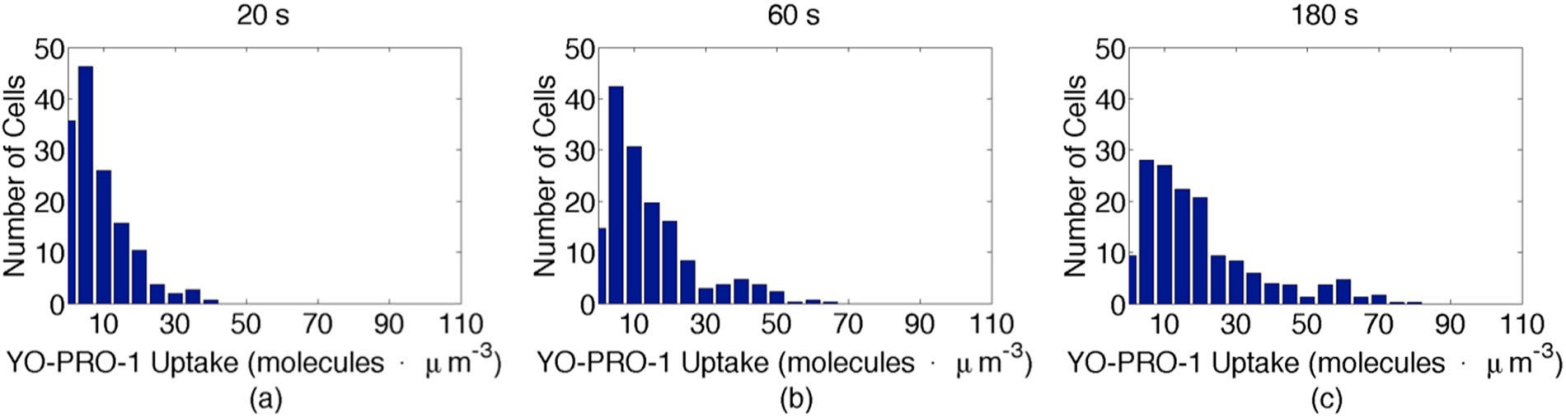
Distribution of YP1 intracellular concentration at (**a**) 20 s (*c*_*average*_ = 6.9 molecules · μm^−3^), (**b**) 60 s (*c*_*average*_ = 18 molecules · μm^−3^), and (**c**) 180 s (*c*_*average*_ = 35 molecules · μm^−3^) after pulse delivery. The distribution broadens with time.

Figure 3 shows the distribution of intracellular concentration of YP1 in the analyzed 157-cell population at 20, 60, and 180 seconds after pulse exposure. The distribution broadens with time, indicating considerable cell-to-cell variation in the rate of uptake. Although the *population average rate* of YP1 uptake decreases over time (Fig. S1), the shape of the distribution of uptake rate does not change significantly (Fig. S2). This means there are no random jumps in the rate of uptake over the time of our observations. Consistent with this, inspection of the rate of uptake of individual cells shows that the cells that have the highest uptake rate earlier in the recording are also the ones that have the highest rate later.

### Cell size does not influence electric-pulse-induced YP1 uptake

The considerable cell-to-cell variation in uptake rate led us to consider factors that could be sources of that variability. One that might be expected to be important is cell size, because of the well-known relation between cell size and the transmembrane voltage induced by an external electric field^39^, which implies that larger cells will be more extensively permeabilized.

**Figure 4:**
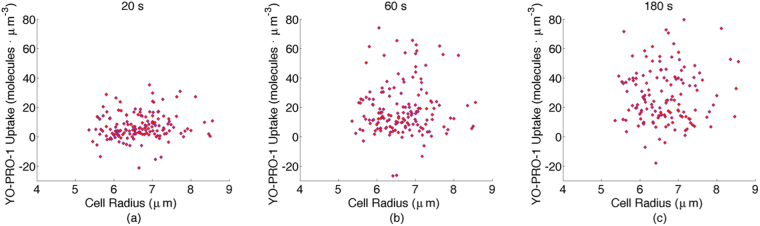
YP1 uptake versus cell radius for 157 cells. Each point indicates a measurement from a single cell. (**a**) 20 s (*R* = 0.057), (**b**) 60 s (*R* = 0.002), and (**c**) 180 s (*R* = 0.028), after pulse delivery.

An examination of YP1 uptake versus cell radius at different time points, however, shows no correlation (Fig. 4), and indeed this is predicted by the “supra-electroporation” model for nanosecond pulse electropermeabilization^40^.

### Molecular simulations of YO-PRO-1 (YP1) transport through electroporated phospholipid bilayers

To compare the electric-pulse-induced molecular uptake of YP1 observed experimentally with the behavior in molecular models of electroporated membranes, we constructed phospholipid bilayer systems with POPC^12^ and added YP1. During equilibration of these systems we noted significant binding of YP1 to POPC. For a 128-POPC system containing 52 YP1 molecules, about half of the YP1 molecules are found at the bilayer interface after equilibration (Fig. S5). We confirmed this unexpected behavior with experimental observations, described below. Similar interfacial YP1 concentrations are found in systems containing approximately 150 mM NaCl or KCl. In systems containing NaCl, YP1 displaces Na^+^ from the bilayer interface (Fig. S6). The binding is mediated primarily by interactions between both positively charged YP1 trimethylammonium and benzoxazole nitrogens and negatively charged lipid phosphate (Fig. S7) or acyl oxygen atoms.

**Figure 5:**
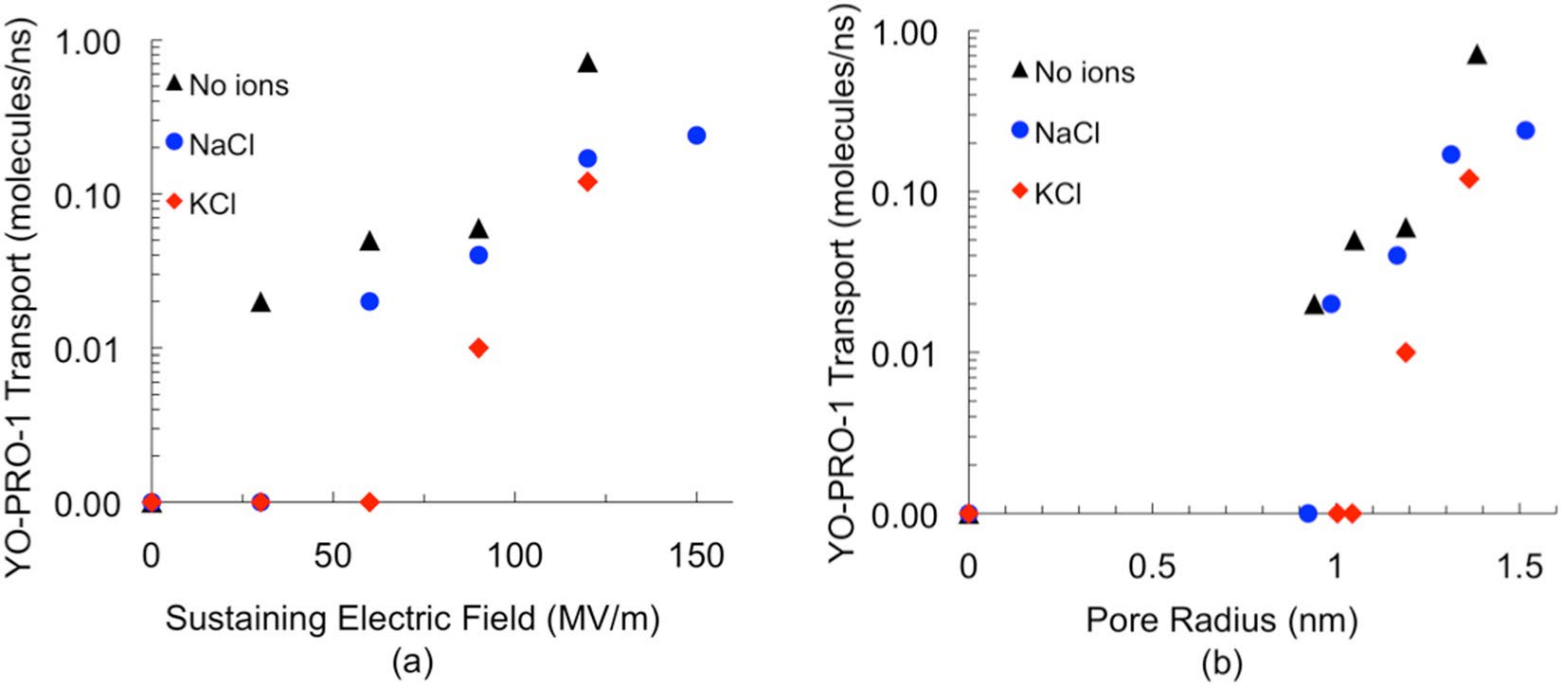
YP1 transport through field-stabilized POPC electropores as a function of (**a**) sustaining electric field and (**b**) pore radius. Black triangles represent systems without electrolytes; blue circles and red diamonds represent systems containing physiological concentrations of NaCl and KCl, respectively.

To observe transport of YP1 through lipid electropores, YP1-POPC systems were porated with a 400 MV/m electric field and then stabilized by reducing the applied electric field to smaller values (120 MV/m, 90 MV/m, 60 MV/m, 30 MV/m, 0 MV/m) for 100 ns, as described previously for POPC systems without YP1^41^. YP1 migrates through the field-stabilized pores in the direction of the electric field, as expected for a molecule with a positive charge. Pore-mediated YP1 transport increases with both electric field magnitude and pore radius, up to about 0.7 YP1/ns at 120 MV/m (Fig. 5). This relationship does not follow a clear polynomial or exponential functional form, and this is not surprising, given the direct dependence of pore radius on stabilizing field in these systems and the fact that, as described below, YP1 traverses the bilayer in association with the pore wall and not as a freely diffusing particle.

**Figure 6:**
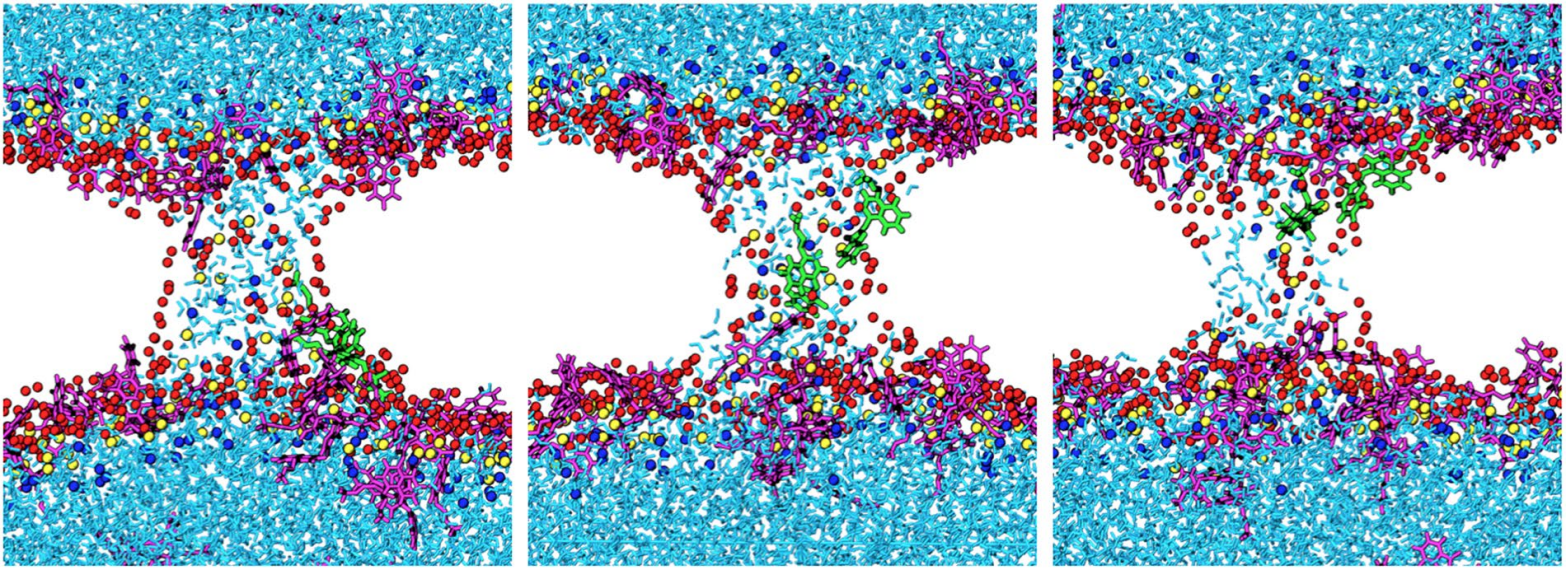
Snapshots of YO-PRO-1 transport through a field-stabilized electropore. Two YP1 molecules (green) are entering the pore at 0 s, halfway across at 50 ns, and merging with the leaflet on the other side at 100 ns.

No transport of free YP1 molecules occurred in the 16 simulations we analyzed. YP1 molecules crossing the bilayer are bound to phospholipid head groups in the pore walls. Even in larger pores, YP1 molecules remain closely associated with the membrane interface as they transit through the pore (Fig. 6). This leads us to predict that YP1 transport rates proportional to the area of the electropore (i.e. follow a second-order polynomial trend in pore radius) will be observed only if and when YP1 binding sites in the pore wall are saturated.

YP1 transport is reduced in the presence of NaCl and KCl, both by mechanical interference from chloride ions moving in the opposite direction and by electrical interactions between the divalent cation YP1 and the monovalent inorganic cations. YP1 transport is particularly small in KCl-containing systems where large amounts of bulk K^+^ and Cl^−^ ions displace YP1 in the electropore interior. In NaCl-containing systems, some Na^+^ is bound to the membrane (Fig. S6), allowing for additional YP1 transport to occur through open electropores.

### YP1 adsorption to cell membranes observed in experiments

**Figure 7:**
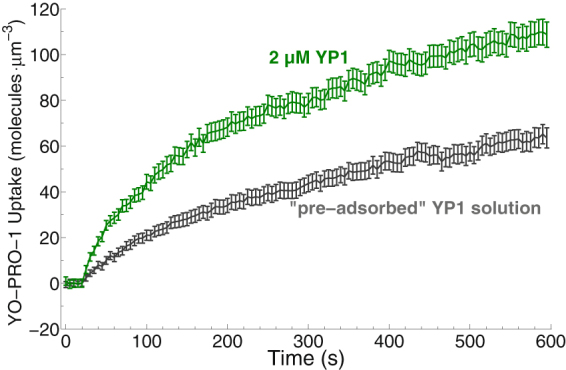
Pulse-induced molecular uptake of YP1 from control medium (2 µM YP1 in RPMI-1640) and from the pre-adsorbed YP1 solution after 5-minute incubation with U-937 cells. The amount of YP1 available for pulse-induced uptake is reduced by about 50% in the medium pre-incubated with U-937 cells. Data are from three separate experiments with 17–18 cells in each experiment.

To validate the observation of membrane binding of YP1 in simulations, we looked for experimentally detectable adsorption of YP1 by cells. For this we compared the uptake in two different solutions: one that contained 2 µM YP1, and one that had contained 2 µM YP1 originally, but then was incubated with a dense cell suspension (1 × 10^7 ^cells/mL) for five minutes before being centrifuged to remove the cells. In other words, the latter of the two solutions lacked the YP1 molecules that were adsorbed by the cells during an incubation of five minutes; we call this the “pre-adsorbed” YP1 solution. In these experiments, the cells were exposed to two 6 ns pulses, 1 ms apart, instead of a single pulse, in order to produce a stronger fluorescence signal and make any difference between the two samples easier to detect. Figure 7 shows that cells quickly adsorb YP1. A five-minute incubation with a dense cell suspension reduces the amount of YP1 remaining in the supernatant after centrifugation to about half the initial value.

## Discussion

### Modeling YO-PRO-1 uptake as diffusive transport through membrane pores

In conventional models for electroporative small molecule entry, YP1 uptake is dominated by diffusion through lipid electropores formed during pulse exposure, and the primary parameters determining YP1 transport are the size and shape of the pores and the solute molecules^15, 37^. This simplified picture of transport is widely accepted and has been used for estimating pore size and number for a given solute size^16, 42^.

These models are consistent with the data in Fig. 2 only if very few pores are formed or the transport of YP1 through individual pores is very slow. Consider the mean molecular uptake over the first 20 s after pulse exposure, when transport is more likely to be dominated by the physical process of diffusion through pores than at later times, when multiple biological stress and damage response mechanisms are active and operating to counter the effects of permeabilization. Assuming that all pores have roughly similar transport properties, then from the uptake rate we can extract the number of pores:

1 $${{N}}_{{pores}}=\frac{{{J}}_{{molecules}{,}{experiment}}\,[{\rm{c}}{\rm{e}}{\rm{l}}{{\rm{l}}}^{-1}]}{{{J}}_{{molecules}{,}{diffusion}{model}}\,[{\rm{p}}{\rm{o}}{\rm{r}}{{\rm{e}}}^{-1}]}=\frac{{{J}}_{{molecules}{,}{experiment}}\,[{\rm{c}}{\rm{e}}{\rm{l}}{{\rm{l}}}^{-1}]}{{J}_{s,p}}$$

*J*_*s*,*p*_ is the diffusive solute flux through a single cylindrical pore,

2 $${J}_{s,p}\,{[\mathrm{pore}}^{-{\rm{1}}}\,\,{s}^{-1}]=HK{J}_{s}$$

where *J*_*s*_ is the diffusive flux due to a concentration gradient (without any interaction of the solute with the pore walls) and *H* and *K* are hindrance and partitioning factors that account for solute-pore interactions^42^.

Leaving the bulk solvent and entering the small volume of the pore is energetically unfavorable for most solutes. The associated partition factor, *K*, is a function of pore radius, solute charge, and transmembrane voltage (Eqs S12–15). Movement of solute molecules in the pore is sterically restricted, represented by the hindrance factor, *H*, a function of solute size and pore radius (Eqs S7–11). Hindrance and partitioning values here are derived as described by Smith^42^, with a transmembrane potential approaching zero (10^−10 ^V) and the charge for YO-PRO-1 set to +2.

*J*_*s*_ is approximated with this expression^43^:

3 $${J}_{s}=\frac{\pi {r}_{p}^{2}\,{D}_{c}\,c}{{d}_{m}+\pi {r}_{p}/2}$$

where *r*_*p*_ and *d*_*m*_ are the dimensions of the pore, *D*_*c*_ is the diffusion coefficient of the solute, and *c* is the extracellular concentration of the solute. Here *d*_*m*_ is set to 4.5 nm. See Supplementary Information for further details.

**Figure 8:**
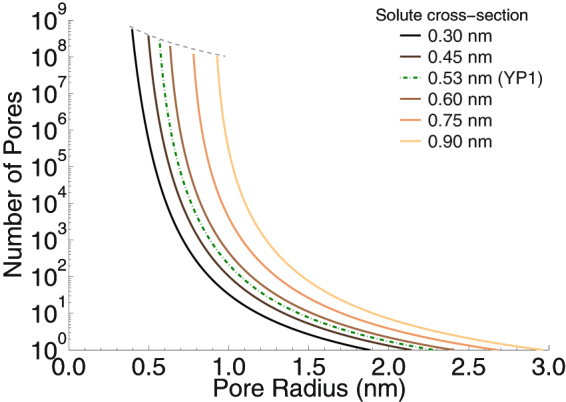
Number of pores needed to transport 180 molecules · s^−1^ · cell^−1^ versus pore radius for different solute sizes in a pore-mediated diffusive transport model. The gradient between extracellular and intracellular concentration were kept constant at 2 µM for all the shown solute sizes. Dashed gray line shows the limit at which total area of pores equals to the area of a whole cell.

With this model for pore-mediated diffusive transport we can estimate the number of molecules transported per pore per second for any given pore radius (Equation 2) and then from Equation 1 calculate the number of pores of a given radius that correspond to our observed molecular transport rate (180 molecules · s^−1^ · cell^−1^; Fig. 2). Figure 8 shows some of these estimates for solutes of different sizes.

For a YO-PRO-1 cross-sectional radius of 0.53 nm^42^, the diffusive transport model tells us that the YO-PRO-1 uptake that we observe requires about 200 pores of radius 1.0 nm (Fig. 8)—roughly 1 (180/200) YO-PRO-1 molecule per pore per second. But note that with this model for diffusion through a pore, very small changes in solute or pore dimensions can modify the transport rate by several orders of magnitude (see Supplementary Information). This sensitivity means that estimating pore size from measured small molecule diffusive transport rates is inherently imprecise. In addition to the technical challenges of measuring transport quantitatively, the pore population in an electroporated cell is not homogeneous and includes pores with time-dependent radii spanning much of the range represented in Fig. 8.

The size of YO-PRO-1-permeant pores has been determined experimentally by two methods. Blocking of pulse-induced osmotic swelling with sucrose suggests that YO-PRO-1 can pass through pores with radii less than 0.45 nm (smaller than the size estimated from the molecular structure, which includes the van der Waals perimeter and does not take into account steric accommodations that may occur during traversal of the pore)^44^. If YO-PRO-1 enters electropermeabilized cells primarily by diffusive transport through pores restricted to this size, the number of pores required would have a total area similar to the area of the cell itself (the upper cut-off of the curves in Fig. 8 as indicated with gray dashed line). However, if the pore population contains in addition to the 0.45 nm pores also just a few hundred pores with radius approaching 1 nm, then our measured transport can be accommodated.

Another estimate of the size of YO-PRO-1-permeant pores, based on comparing electroporation-induced uptake of YO-PRO-1 and propidium dyes, gives a radius of 0.7 nm^16^. This value fits more comfortably within the diffusive transport range of pore numbers and sizes shown in Fig. 8 (7 × 10^4^ pores with radius 0.7 nm would be sufficient for our observed YO-PRO-1 uptake). Note that a change in average pore size from 0.45 nm to 0.7 nm corresponds to an increase of two orders of magnitude in the transport predicted by the pore diffusion model. The large uncertainties involved in these estimates, however, and the cell-to-cell variation in measured uptake, mean that values for pore radius within the sub-nanometer range cannot be excluded. These numbers should be taken not as fixed, hard dimensions, but rather as indicators of boundaries for pore size, to be applied to the still poorly characterized distribution of radii within a pore population.

### Electro-transport of membrane-bound YP1

Our molecular dynamics simulations suggest that a significant component of YP1 transport through lipid electropores involves YP1 molecules bound to the phospholipid bilayer, which is quite different from the diffusion of solvated molecules through openings in the membrane that dominates current models. Although the molecular dynamics simulations presented here can be interpreted only qualitatively until the YO-PRO-1 model can be validated more extensively, some conclusions can be drawn from these preliminary results.

First, as confirmed experimentally, YP1 binds to cell membranes. Binding interactions between transported species and the cell membrane must be quantified and taken into account in models of the electroporative transport of small-molecule fluorescent dyes into cells.

Second, YP1 transport across the membrane in our molecular models is not simple diffusion or electrophoretic drift through the medium filling the pore but rather an interface phenomenon involving interactions of YP1 and the phospholipid head groups forming the wall of the pore. Similar observations have been reported for larger molecules (siRNA and the peptide CM_18_-Tat_11_) in previous molecular dynamics studies^45, 46^. Nevertheless, the rate of movement of YP1 across the membrane in the simulation is not inconsistent with the experimental data if, for example, we assume a non-zero post-pulse membrane potential.

At the pore-sustaining electric fields used here, which are not much greater than the field arising from the unperturbed resting potential of the cell membrane (80 mV across 4 nm is 20 MV/m), the rate of YP1 transport through the pore is approximately 0.1 YP1 · ns^−1^ for pores with radii just above 1.0 nm (Fig. 5). Even if we reduce this by a factor of 10, to represent the lower post-pulse transmembrane potential, the simulated *single-pore transport rate*, 1 × 10^7^ YP1 · s^−1^, is several orders of magnitude greater than the mean rate per cell of YP1 transport experimentally observed and reported here. However, note that the concentration of YP1 in these simulations (120 mM) is also quite high. Taking this factor into account, a single 1 nm electropore will transport on the order of 200 YP1 · s^−1^, which is roughly the measured transport for an *entire* permeabilized cell. This estimate of the transport rate could be further reduced if the rate of dissociation from the membrane is slower than the rate of translocation through the pore, resulting in a requirement for a higher number of pores.

Pores that are slightly smaller, however, may have YP1 transport properties that are more compatible with our experimental observations. Because our YP1 transport simulation times are of practical necessity very short (100 ns), we cannot accurately monitor YP1 transport in the model when the pore radius is 1 nm or less (Fig. 5)—the number of molecules crossing the membrane through a single pore is less than one in 100 ns. It is not unreasonable to speculate, however, that YP1 transport rates for simulated pores in this size range may be compatible with rates extracted from the diffusion model. For example, from Fig. 8, about 200 pores with radius 1 nm or 800 pores with radius 0.9 nm or 4600 pores with 0.8 nm radius would account for the YP1 transport we observe.

### Boundaries on mechanistic models for electroporative transport of small molecules into cells

Although the preceding analysis indicates the possibility of a formal mapping of small molecule electroporation transport data onto molecular models and geometric models of diffusive influx through pores, we see several difficulties with this approach.

First, the transport-related properties of any given pore in the pore diffusion models are based on a simple geometry that evolves only in radius space (even in the most developed models), and there is no representation of non-mechanical interactions of solute molecules with the components of the pores. This results in an inadequate representation of the transport process itself, as our molecular simulations indicate.

Even for a small, simple molecule like YO-PRO-1, transport through a lipid pore involves more than geometry and hydrodynamics. We have shown here, experimentally and in molecular simulations, that YO-PRO-1 crosses a porated membrane not as a freely diffusing solute molecule but rather at least in part in a tightly bound association with the phospholipid interface. YO-PRO-1 entry into the cell may be better represented as a multi-step process, like that observed for DNA^34^, that is facilitated by electropore formation, but which cannot be described simply as a passage of the molecules through pores.

Second, restricting transport to pore-mediated diffusive migration through simple, membrane-spanning openings means that permeabilizing structures other than lipid pores (for example, electromodulated protein channels^31^, scrambled, destabilized, peroxidized lipid regions^24^, obstructed pores^47^, small-molecule-permeant protein channels like P2X_7_, TRPA1, Panx1^48–50^, endocytotic and exocytotic vesicles, etc.) are not represented.

Third, lipid pore lifetimes in molecular models^9, 12^, and in artificial membranes and vesicles^51, 52^, are much too short to account for permeabilization in living cells, which lasts for minutes. Although recent models for post-electroporation transport through lipid pores have begun to include pore populations with longer lifetimes^53^, there is no substantiated experimental evidence for a stable state for simple lipid pores over the many minutes of post-permeabilization transport reported in many studies of electroporated cells^21, 26^, including now in this report, after the minimal perturbation of a single, 6 ns pulse exposure. One possible mechanism for resolving this apparent discrepancy between lipid bilayers and cell membranes, at least in part, lies in the recovery of the cell’s transmembrane potential. If this happens quickly^21^, it could contribute to the stabilization of lipid pores formed during pulse application^41, 54^. Until the evidence for this is stronger, however, we must expect that most long-lived membrane permeabilizing structures are not simple lipid electropores.

Finally, and perhaps most importantly, models of electroporation based on pore-mediated transport ignore cellular *responses* to membrane permeabilization. This includes not only dynamic modifications to the properties of the lipid bilayer and the lipid pore population, but also transport-related processes associated with the reactions of the cell to the stress and damage resulting from membrane barrier disruption (redistribution of anionic phospholipids, recovery from Ca^2+^ influx and K^+^ and ATP efflux, restoration of ion concentration gradients and membrane resting potential, volume regulation, and membrane repair).

Starting from a quantitative, experimental determination of YP1 uptake into cells permeabilized with a very short (6 ns) pulsed electric field, we have identified possible points of intersection with small-molecule transport models based on pore-mediated diffusion and molecular mechanics. Whether the intersection noted above around *r*_*p*_ = 1 nm corresponds to a genuine alignment of the models with the experimental data can be determined by evaluating small-molecule transport experimentally with solutes other than YO-PRO-1, with different sizes and different chemical and electrical properties, and by increasing the resolution of the molecular dynamics simulations by running them for longer times. For example, measured values for transport of the fluorescent dyes propidium, a divalent cation like YO-PRO-1 but a somewhat larger molecule, and calcein, a similar-sized divalent anion, can be compared to the predictions of the pore-mediated diffusion model for *r*_*p*_ = 1 nm. Given sufficient computing resources, the number of YO-PRO-1 molecules transported in molecular simulations of 1 nm (or smaller) pores can be counted for much longer times, 1 s or more, to determine the transport rate more accurately.

The experimental data provides a reference point for evaluating the validity of the models, and for establishing limits on the parameters which set the range of predictions from the models. The molecular model is more restrictive because it is grounded in the physical properties of the membrane, but it is also dependent on how accurately those properties are represented. The pore-mediated diffusion model is much more loosely constrained, because it is abstract and open-ended (parameters may be added or re-defined, to represent greater and greater complexity).

To address the limitations of the pore-mediated diffusive transport models discussed above, we must begin the construction of an expanded and extended model that *includes* diffusion through lipid pores, but *as one among several* transport mechanisms in electropermeabilized cells. “Electropermeabilization of membranes can no longer be described simply as ‘punching holes’ in a lipid bilayer as described by the electroporation hypothesis”^55^.

### The electropermeabilized cell and the electropermeome

Cell homeostasis is disrupted by electroporation in many ways: high intracellular Ca^2+^, depletion of ATP and K^+^, osmotic imbalance, electrical depolarization, scrambling of bilayer asymmetry, and other membrane disorganization resulting from water infiltration and pore formation. These assaults, coming all at once, present an abrupt and dangerous stress to cellular systems. The leaks must be closed and the permeabilizing structures removed or repaired, and the many ensuing physiological disturbances must be corrected. The cell must respond immediately and effectively, or it will die.

Electroporation models that are restricted to pore-associated diffusion ignore these stress and damage responses to membrane permeabilization and do not take into account the multiple transmembrane pathways present in the electropermeabilized cell. To be accurate, predictive, and robust, an electroporation model must represent not only the initial permeabilized state, but all of the subsequent, permeabilization-induced transport structures and repair and restoration processes, which, taken together, we call the *electropermeome*.

Our findings raise questions regarding how well current electroporation models represent the set of permeabilizing structures and processes (the electropermeome) that contribute to the long and slow uptake of molecules like YP1 after a permeabilizing event lasting only 6 ns. We point to previously published evidence for some of these structures and processes, and here we identify several components of the electropermeome that must be included in a comprehensive model.

1. Physical structures: The first set is comprised of the physical structures formed as a consequence of the immediate interaction of the electric field with membrane biomolecular assemblies: simple lipid electropores and pores with cytoskeletal constraints^56^ or obstructions^47^, electroconformationally altered membrane proteins^31, 57^, regions of lipid scrambling^58^ or peroxidation^24, 25^, and polynucleotide-membrane complexes^34^.
2. Biological processes: The electropermeome includes also the post-permeabilization processes that are part of the cellular stress and damage response: volume regulation and pressure release triggered by osmotic swelling^44^, sodium-potassium and calcium ion ATP-dependent pump activity following membrane depolarization and loss of ion concentration gradients^27, 30^, and membrane repair^59^. These processes take place in a compromised metabolic environment. ATP, the cell’s primary energy currency, is leaking into the medium just when it is needed for calcium and sodium-potassium pumps and membrane restructuring and repair^26^. And for some kinds of electric pulse exposures, the mitochondria themselves are permeabilized, with associated loss of the proton gradient essential for aerobic glycolysis^60^. A model that accurately predicts the time course of recovery from the electropermeabilized state must incorporate these considerations of metabolic balances and reserves.
3. Other potential contributors: ATP efflux activates additional potential components of the electropermeome, purinergic receptor channels like P2X_7_, which is associated with cationic small molecule uptake^48^, including YO-PRO-1. Blebbing, like that observed after permeabilizing pulse exposure, is also associated with P2X_7_ channel activation^61^. Other membrane proteins which may become part of the electropermeome include TRP channels, some of which are voltage-, mechano-, or temperature-sensitive^62, 63^, and which can be permeant to cationic small molecules like YO-PRO-1 and NMDG^49^, voltage-gated connexin hemichannels^64^, and ATP- and YO-PRO-1-permeant pannexin channels^50^.

A model of electroporation cannot be broadly and quantitatively predictive without representing the entire dynamic, post-pulse, biological landscape of transport after membrane electropermeabilization.

### Summary

We quantify the uptake of the normally impermeant small molecule fluorescent dye YO-PRO-1 into living cells after a single 6 ns, permeabilizing electric pulse (20 MV/m) with 2 µM YO-PRO-1 in the external medium. The rate of uptake for the first 20 seconds is 180 molecules · cell^−1^ · s^−1^. After 3 minutes the uptake has slowed to 26 molecules · cell^−1^ · s^−1^, and it continues without further slowing for at least 7 minutes. These rates of transport intersect tangentially those predicted by standard electroporation models, but precise alignment of experiment and model is dependent on the validity of the assumption that transport after electropermeabilization is dominated by diffusion through lipid pores. The long duration of the permeabilized state after even a single, 6 ns permeabilizing pulse, and the evidence from experiment and from molecular simulations of significant binding of YO-PRO-1 to the membrane, even during transport, challenges this assumption and indicates that diffusion through transmembrane aqueous pores may not be the primary transport mechanism for small molecule fluorescent dye indicators of membrane permeabilization. Electropermeabilization-induced transport is much more complex than pore-mediated diffusion. To be predictive and quantitative, models must represent all of the transport-related structures and processes in the electroporated cell (the electropermeome).

## Materials and Methods

### Cells

U-937 (human histiocytic lymphoma monocyte; ATCC CRL-1593.2) cells^65^ were cultured in RPMI-1640 medium (Corning® glutagro™ 10-104-CV) with 10% fetal bovine serum (Corning, 35-010-CV) and 1% penicillin/streptomycin (10000 U/mL penicillin and 10 mg/mL streptomycin) at 37 °C in a humidified, 5% CO_2_ atmosphere. For microscope observations cells were washed and suspended at approximately 5 × 10^5 ^cells/mL in fresh medium containing 2 µM YO-PRO-1 (Molecular Probes) for experiments.

### Pulsed Electric Field Exposure

**Figure 9:**
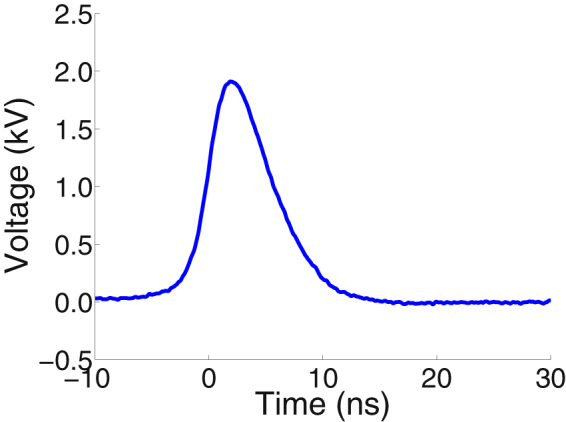
Typical 6 ns waveform. Waveform recorded as it was applied during the experiments.

Single 6 ns FWHM, 2 kV pulses from an FID pulse generator (FPG 10-10 NK) were delivered to cells in cover glass chambers (Thermo Scientific™ Nunc™ Lab-Tek™ II) through parallel tungsten wire electrodes with a separation of 100 µm, positioned in the chamber with a micromanipulator^66^. Pulses of amplitude 2 kV and duration 6 ns FWHM waveforms were measured with an oscilloscope (Fig. 9). For adsorption experiments two pulses were delivered at 1 kHz repetition rate.

### Adsorption Experiments

Cells were suspended in RPMI-1640 medium containing 2 µM YO-PRO-1 at 1 × 10^7 ^cells/mL in a 37 °C, humidified, 5% CO_2_ atmosphere. After 5 minutes the cells were pelleted by centrifugation at 455 g for 2 minutes, and the supernatant was removed. Experiments were performed with either untreated RPMI-1640 containing 2 µM YO-PRO-1 or the supernatant obtained as described.

### Imaging

Laser scanning confocal fluorescence microscope images were captured (Leica TCS SP8) every five seconds for ten minutes (120 frames) from cell suspensions at room temperature in ambient atmosphere on the microscope stage. Cells were exposed to a single, 6 ns, 20 MV/m electric pulse 22 seconds after the start of the recording.

### Image Processing

Approximately half of the cells visible in the microscope field between the electrodes were randomly selected (MATLAB random function) for fluorescence photometric image analysis before each pulse exposure. Fluorescence intensities of each region of interest were extracted using custom MATLAB routines. The following built-in MATLAB functions were used in custom image processing routines.: ‘imroi’, for manually choosing regions of interest; ‘random’, for creating a uniform probability distribution function that generates random numbers within a given set from which cells are selected for analysis; ‘regionprops’, for evaluating geometric properties of regions of interest.

### Calibration

The procedure for correlating YO-PRO-1 fluorescence to molar concentration closely follows a method previously described^16^. Dense lysates were created from U-937 cells (8 × 10^7^ cells/mL) by adding 0.1% Triton-x100 and then sonicating for 2 minutes with a Misonix Sonicator S-4000 sonicator, 1 second alternating on-off cycle, amplitude 20.

**Figure 10:**
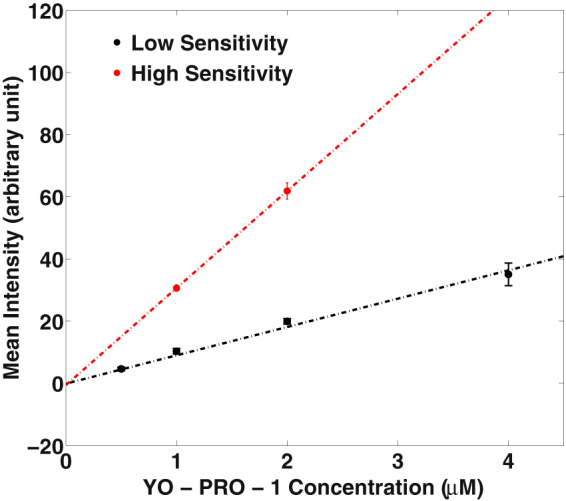
Calibration curve for YO-PRO-1 with Leica TCS SP8 confocal microscope using two different imaging settings. Error bars are standard deviation.

The fluorescence from samples of this lysate containing fixed concentrations of YO-PRO-1 (YP1) was captured in confocal slices 10 µm above the bottom of 8-well cover glass chambers. Calibration curves were generated for two sets of imaging parameters, one for fast, less sensitive measurements, one for greater sensitivity (Fig. 10). Each point on the curves represents measurements taken in triplicate from three separate preparations.

Slope of the calibration curve is used to extract number of molecules per micrometer cube for a given set of imaging parameters from arbitrary fluorescence units according to equation below.

4 $$Influx\,({\rm{molecules}}/{{\rm{\mu }}{\rm{m}}}^{3})=\frac{\begin{matrix}Fluorescence\,({\rm{arbitrary}}\,{\rm{units}})\times {N}_{A}\,(\mathrm{molecules}/\mathrm{mole})\times 1\,(\mathrm{mole}/\mathrm{liter}/{\rm{M}})\end{matrix}}{Calibration\,curve\,slope\,({\rm{arbitrary}}\,\,\mathrm{units}/{\rm{M}})\times {10}^{15}\,({{\rm{\mu }}{\rm{m}}}^{3}/\mathrm{liter})}$$

All of the results presented here are taken from measurements using the high-sensitivity parameters, which permit imaging at 5 s/frame.

### Molecular Dynamics Simulations

Simulations were performed using GROMACS version 4.6.5^67^. 1-palmitoyl-2-oleoyl-*sn*-glycero-3-phosphotidylcholine (POPC) topologies, obtained from D. Peter Tieleman, utilize the OPLS/Berger force field^68^. They were hydrated with 70 explicit SPC/E rigid water molecules per lipid. This produced a box size of roughly 6.5 × 6.5 × 10 nm^3^, containing 128 lipids per bilayer (64 lipids/leaflet). YO-PRO-1 (YP1) topologies were obtained by first utilizing the PRODRG server^69^ to obtain Lennard-Jones constants and partial charge assignments. Subsequently, the partial charge distribution for the YP1 choline group was modified to match the POPC choline distribution, while the nitrogen charge group on the YP1 oxazole ring was empirically distributed to reproduce the experimental YP1 electrophoretic mobility, which was measured at 2.8 × 10^−8 ^m^2^/V-s in simulations. As a result, YP1 held a net charge of +2, requiring the insertion of two chloride counter ions to neutralize the net charge of the system. Bilayers were equilibrated for 100 ns in an NPT ensemble until they exhibited a constant area per lipid at 310 K, using the velocity rescaling thermostat of Bussi *et al*.^70^, and the weakly coupled Berendsen barostat^71^ that maintained 1 bar of isotropic pressure under an isothermal compressibility of 4.5 × 10^−5 ^bar^−1^. Periodic boundary conditions were implemented in all directions to mitigate system size effects and reduce the time required for computation. A leapfrog algorithm was utilized in order to integrate Newton’s equations of motion at an integration time step of 2 fs. YP1 and POPC molecular bonds were constrained using the LINCS algorithm^72^, while water bonds were constrained using the SETTLE algorithm^73^. Short-range electrostatic and Lennard-Jones forces were truncated at 1 nm, where long-range interactions were turned on and tabulated using the Particle Mesh Ewald (PME) algorithm^74^, which utilizes Fast Fourier Transforms. When applicable, 40 NaCl or 22 KCl were then inserted into bilayer systems, as in previous studies, and equilibration was continued until ion binding to the membrane interface converged. Following this, 51 YP1 molecules were added. After convergence of YP1 binding to the bilayer, 25 YP1 molecules remained free in the bulk solvent (120 mM). Membrane electropores were then created and expanded by applying field magnitudes of 400 MV/m to the bilayer normal^12^, followed by the application of smaller, pore-sustaining electric fields^41^. Pore radius measurements were extracted using a previously described method^12^. Molecular graphics were generated using Visual Molecular Dynamics (VMD 1.9.1)^75^.

## Electronic supplementary material

Supplementary Information
