# Supplementary material for: Quantitative Limits on Small Molecule Transport via the Electropermeome — Measuring and Modeling Single Nanosecond Perturbations
Source: Sci Rep. 2017 Mar 3;7:57. doi: 10.1038/s41598-017-00092-0 (PMC5428338; doi:10.1038/s41598-017-00092-0)
Supplement: Supplementary file 1 — Supplementary Information [file 41598_2017_92_MOESM1_ESM.pdf]

Quantitative Limits on Small Molecule Transport via the Electropermeome — Measuring and Modeling Single Nanosecond Perturbations

Esin B. Sözer[1], Zachary A. Levine[2], and P. Thomas Vernier[1]

[1]Frank Reidy Research Center for Bioelectrics, Old Dominion University, 23508, Norfolk, VA USA
[2]Department of Physics, Department of Chemistry and Biochemistry, University of California Santa Barbara, Santa Barbara, CA 93106 USA
Email: pvernier@odu.edu

Supplementary Information

Rate of Uptake

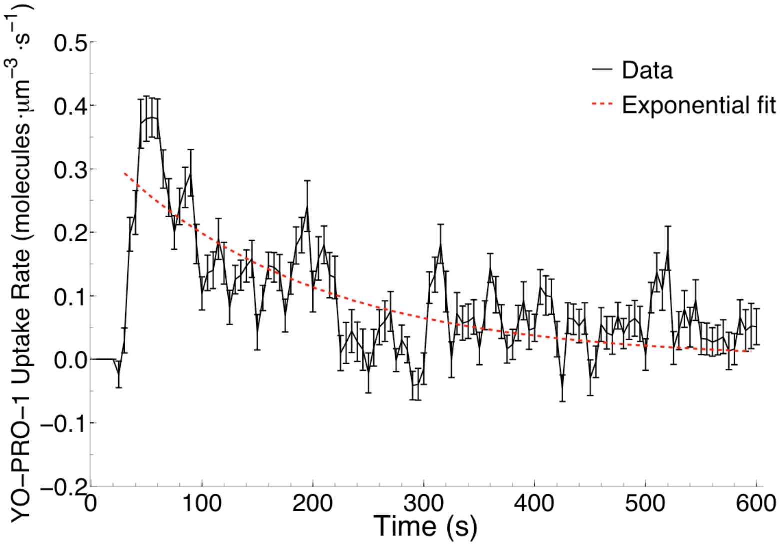

Figure S1. Rate of YP1 uptake into cells over time. Data is filtered with a 20 second moving difference filter[1]. The best exponential fit gives a decay time constant of 180 s.

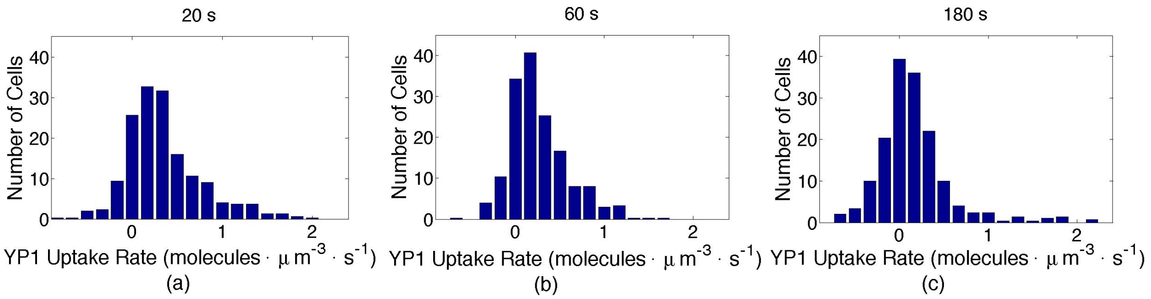

Figure S2. Distributions of YP1 uptake rate at three different times (20 s, 60 s, 180 s) after pulse exposure (n = 157). Data from 15 s windows centered on each of the three time points were averaged and binned (bin size 0.08 molecules · $\mu m^{-3} \cdot s^{-1}$). Peak values: (a) 20 s, 0.33 molecules/$\mu m^3 \cdot s^{-1}$, (b) 60 s, 0.27 molecules/$\mu m^3 \cdot s^{-1}$, (c) 180 s, 0.15 molecules/$\mu m^3 \cdot s^{-1}$.

Rate of uptake is another way to look at the data reported in Figure 2. In Figure S1 we plotted time derivative of the same data, showing the decrease in rate of uptake with time. Rate of uptake at time point *t*, R_t is filtered in this plot using moving average difference filter, such that

$$R_t = \frac{\varphi_t - \varphi_{t-4}}{4\Delta t} \tag{S1}$$

where φ_t is the uptake at point *t* and Δt is the timestep of the recording.

Distribution plots of rate of uptake in Figure S2 show that the shape of the distribution is maintained, meaning there are no irregular jumps in rate of uptake during the time course causing broadening or narrowing of the distribution. Moreover, consistent with maintained width of the distribution, an inspection of changes in rate of uptake for individual cells showed that as the population average is decreasing with time, cell-to-cell variation of rates of uptake is maintained. In other words, cells with higher rate of uptake at 20 seconds are also the same ones with higher rate of uptake at 180 seconds after the field exposure.

YO-PRO-1 Uptake versus cell location with respect to electrodes

We looked at correlation of cell location between the electrodes to the total uptake of YP1 molecule at different time points (Fig. S3) and found no direct correlation. This observation is consistent with the uniform electric field distribution we expect to have between the parallel wire electrodes based on electrostatic simulations of the electrode assembly reported in Wu et al.[2].

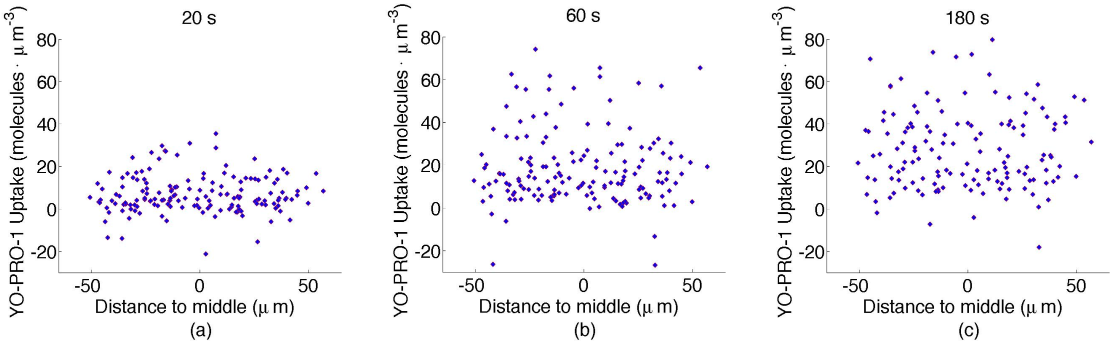

Figure S3. Total YP1 uptake versus cell location for 157 cells. Each point indicates a single measurement of a single cell. 0 μm is the midpoint between the electrodes. (a) 20 s (R = 0.025), (b) 60 s (R = -0.022), and (c) 180 s (R = -0.052) after pulse delivery.

Diffusion Coefficient for YO-PRO-1

An experimental value for the diffusion coefficient of YO-PRO-1 (YP1) was not found in the literature, instead we used an estimate based on the geometrical properties of the YP1 molecule. For a spherical particle with a well-established diffusion coefficient we used the sodium ion as a reference. From its diffusion coefficient (D_{Na}), and radius (r_{Na}), we

can extract an estimate for the diffusion coefficient of an ellipsoid particle with longer radius ($l_{YP1}/2$) and shorter radius (r_{YP1}).

.

Diffusion coefficient (D) and friction coefficient (ξ) are related[3]:

$$D = \frac{kT}{\xi} \tag{S2}$$

where k, and T are Boltzmann's constant, and absolute temperature. For a spherical particle like the sodium ion the friction coefficient is

$$\xi_{\text{Na}} = 6\pi\eta r_{\text{Na}} \tag{S3}$$

where η is viscosity of the solvent. For ellipsoid solutes moving sideways with longer radius ($l_{YP1}/2$) and shorter radius of (r_{YP1}) the friction coefficient is

$$\xi_{YP1} = \frac{4\pi\eta l_{YP1}}{\ln(l_{YP1}/r_{YP1}) + 1/2} \tag{S4}$$

Using S2, S3, and S4, we can get diffusion coefficient for YP1:

$$D_{YP1} = \frac{6\, D_{Na}\, r_{Na}}{4\, l_{YP1}} \times \left[\ln\left(\frac{l_{YP1}}{r_{YP1}} \right) + 1/2 \right] \tag{S5}$$

This calculation gives a diffusion coefficient for YP1 equal to 4.50 $\times 10^{-10}$ m^2/s (l_{YP1} = 1.71 nm, r_{YP1} = 0.53 nm)[4]

Sensitivity of diffusive uptake calculations to hindrance and partitioning effects

As described under section "Modeling YO-PRO-1 uptake as diffusive transport through membrane pores", $J_{s,p}$, diffusive uptake through a single cylindrical pore can be described as:

$$J_{s,p}\ [\text{pore}^{-1}\ s^{-1}] = HKJ_s \quad , \tag{S6}$$

where J_s is the diffusive uptake due to a concentration gradient (without any interaction of the solute with the pore walls) and H and K are hindrance and partitioning factors that account for solute-pore interactions[4].

Hindrance (H) arises from two factors, decreased effective area when solute is passing through the pore (f_A), and the drag exerted on the solute by the pore walls (f_D), as developed by Bungay and Brenner[5]. For a detailed explanation of the calculation of each factor please refer to Smith[4]. Here we give the specific equations that we used for calculations that generated Fig. 8 of the main text.

$$H = \hat{f}_D \, f_A \tag{S7}$$

where effective area f_A is given by

$$f_A = (1 - \lambda)^2 \tag{S8}$$

with $\lambda = r_s/r_p$ where r_p is the radius of the pore, and $\hat{f}_D$ is the drag factor (f_D) modified for a cylindrical solute according to

$$\hat{f}_D = \frac{f_D}{f_D + (1 - f_D)\dfrac{l_s}{2 r_s}} \tag{S9}$$

where l_s and r_s are solute dimensions.

The drag term f_D is calculated according to Bungay and Brenner[5]:

$$f_t = \frac{9}{4}\pi^2 \sqrt{2}\,(1 - \lambda)^{-5/2}(1 + a_1(1 - \lambda) + a_2(1 - \lambda)^2) + a_3 + a_4 \lambda + a_5 \lambda^2 + a_6 \lambda^3 + a_7 \lambda^4 \tag{S10}$$

$$f_D = \frac{6\pi}{f_t} \tag{S11}$$

with constants $a_1 = -1.2167$, $a_2 = 1.5336$, $a_3 = -22.5083$, $a_4 = -5.6117$, $a_5 = -0.3363$, $a_6 = -1.216$, $a_7 = 1.647$.

Partitioning (K) accounts for the energetic cost of moving a charged solute from a high dielectric constant medium to the low dielectric constant interior of a lipid bilayer.

$$K = \frac{A}{B \exp(\gamma) - C} \tag{S12}$$

where A, B, C are factors given below and

$$\gamma = \frac{q_e z_s V_m}{kT} \tag{S13}$$

where z_s is the valence of the solute, q_e is the elementary charge, V_m is the membrane potential, k is the Boltzmann constant and T is the absolute temperature.

$$A = \exp(\gamma - 1)$$
$$B = \frac{(w_0 \exp(w_0 - n\gamma) - n\gamma)}{w_0 - n\gamma}$$
$$C = \frac{(w_0 \exp(w_0 + n\gamma) + n\gamma)}{w_0 + n\gamma} \tag{S14}$$

with $n = 0.25$, and the Born energy

$$w_0 = \frac{5.3643 \times (z_s q_e)^2}{kT} \times (r_{pore})^{-1.803} \tag{S15}$$

In this calculation, small changes in pore radius and solute radius can lead to large changes in diffusive uptake because of how hindrance and partition factors change with pore size. Figure S4 shows hindrance and partitioning for a solute with radius of $r_s = 0.53$ nm together with the combined effect of these factors on total transport is in red. Note that Fig. S4 is a semilog plot, and when the pore size approaches to solute size on the left hand side of the plot, both factors decrease dramatically with a small change in pore size. For example, a 0.05 nm change in pore size— a fraction of the size of a water molecule — from 0.6 nm to 0.65 nm causes, more than a tenfold change in the transport rate, while a change from 0.9 nm to 1.0 nm in pore size ends up in a three-fold change in the transport rate for a solute with a cross-sectional radius of 0.53 nm, approximately the size of YO-PRO-1. This sensitivity in calculations is important to note since, up to now, there is no evidence of such sensitivity experimentally.

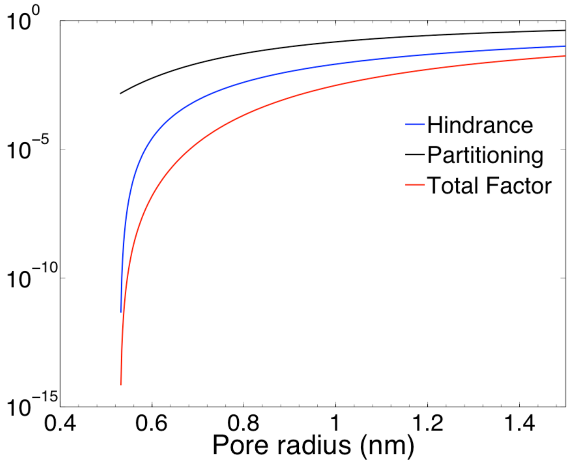

Figure S4. Hindrance and partitioning as a function of pore radius, for a solute of radius r_s = 0.53 nm.

YO-PRO-1 Binding to POPC in molecular dynamics simulations

Figure S5 shows the total number of interfacial (bound) and bulk (free) YP1 molecules in a 128-POPC system containing 52 YP1 molecules as a function of time.

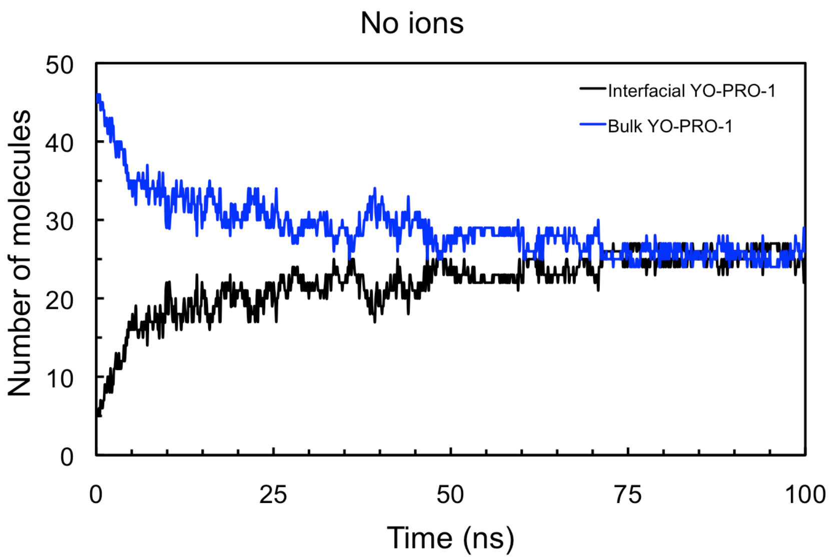

Figure S5. Redistribution of YP1 from the bulk solution to the bilayer interface

Similar interfacial YP1 concentrations are found in systems containing NaCl or KCl. In systems containing NaCl, YP1 displaces Na^+ from the bilayer interface (Fig. S6).

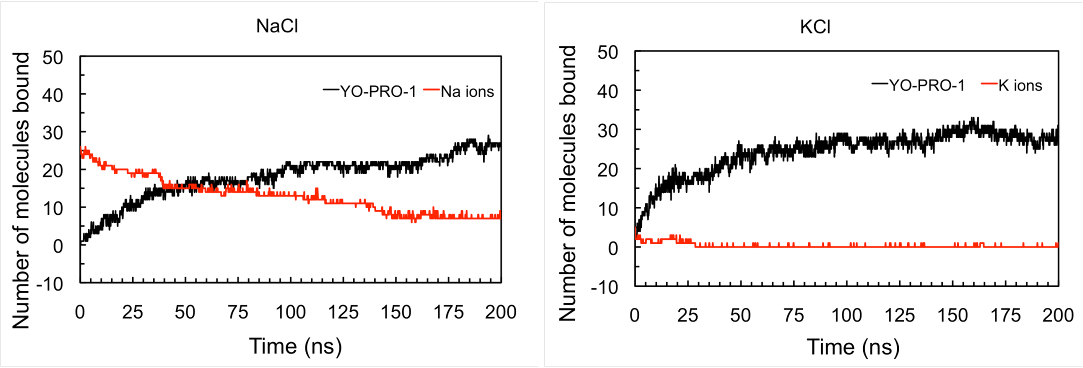

Figure S6. Displacement of interfacial Na^+ by YP1. K^+ ions do not affect the binding behavior of YO-PRO-1

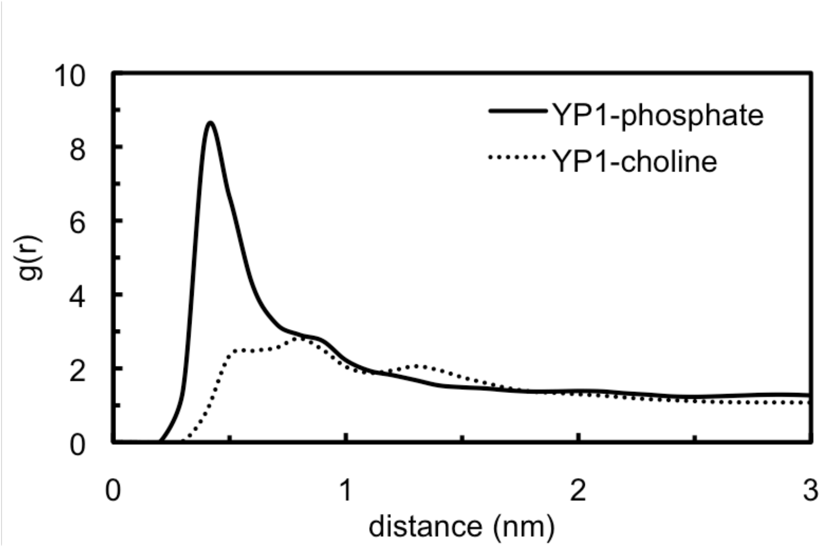

Figure S7. Radial distribution function of YP1 showing strong binding affinity between positively charged YP1 nitrogens and negatively charged POPC phosphate residues, while interactions with positively charged choline groups were three times less likely.

References

1. Strang, G. & Nguyen, T. *Wavelets and filter banks*. (Wellesley-Cambridge Press, 1996).
2. Wu, Y.-H. *et al*. Moveable wire electrode microchamber for nanosecond pulsed electric-field delivery. *IEEE Trans. Biomed. Eng.* **60,** 489–496 (2013).
3. Dill, K. & Bromberg, S. *Molecular driving forces: statistical thermodynamics in biology, chemistry, physics, and nanoscience*. (Garland Science, 2010).
4. Smith, K. C. A Unified Model of Electroporation and Molecular Transport. (2011).
5. Bungay, P. M. & Brenner, H. The motion of a closely-fitting sphere in a fluid-filled tube. *Int. J. Multiph. Flow* **1,** 25–56 (1973).
